# Complete mesocolic excision for right hemicolectomy: an updated systematic review and meta-analysis

**DOI:** 10.1007/s10151-023-02853-8

**Published:** 2023-08-26

**Authors:** G. De Lange, J. Davies, C. Toso, G. Meurette, F. Ris, J. Meyer

**Affiliations:** 1 Medical School, University of Geneva, Rue Michel-Servet 1, 1206 Geneva, Switzerland; 2 Division of Digestive Surgery, University Hospitals of Geneva, Rue Gabrielle-Perret-Gentil 4, 1211 Geneva 14, Switzerland; 3 Cambridge Colorectal Unit, Addenbrooke’s Hospital, Cambridge University Hospitals NHS Foundation Trust, Cambridge, UK; 4 University of Cambridge, Cambridge, UK

**Keywords:** Complete mesocolic excision, D3 lymphadenectomy, Colon, Cancer, Surgery

## Abstract

**Purpose:**

Complete mesocolic excision improves lymphadenectomy for right hemicolectomy and respects the embryological planes. However, its effect on cancer-free and overall survival is questioned. Therefore, we aimed to determine the potential benefits of the technique by performing a systematic review of the literature and meta-analysis of the available evidence.

**Methods:**

Web of Science, PubMed/Medline, and Embase were searched on February 22, 2023. Original studies on short- and long-term oncological outcomes of adult patients undergoing right hemicolectomy with complete mesocolic excision as a treatment for primary colon cancer were considered for inclusion. Outcomes were extracted and pooled using a model with random effects.

**Results:**

A total of 586 publications were identified through database searching, and 18 from citation searching. Exclusion of 552 articles left 24 articles for inclusion. Meta-analysis showed that complete mesocolic excision increased the lymph node harvest (5 studies, 1479 patients, MD 9.62, 95% CI 5.83–13.41, *p* > 0.0001, *I*^2^ 84%), 5-year overall survival (5 studies, 2381 patients, OR 1.88, 95% CI 1.14–3.09, *p* = 0.01, *I*^2^ 66%), 5-year disease-free survival (4 studies, 1376 patients, OR 2.21, 95% CI 1.51–3.23, *p* < 0.0001, *I*^2^ 0%) and decreased the incidence of local recurrence (4 studies, 818 patients, OR 0.27, 95% CI 0.09–0.79, *p* = 0.02, *I*^2^ 0%) when compared to standard right hemicolectomy. Perioperative morbidity was similar between the techniques (8 studies, 3899 patients, OR 1.04, 95% CI 0.89–1.22, *p* = 0.97, *I*^2^ 0%).

**Conclusion:**

Meta-analysis of observational and randomised studies showed that right hemicolectomy with complete mesocolic excision for primary right colon cancer improves oncologic results without increasing morbidity/mortality. These results need to be confirmed by high-quality evidence and randomised trials in selected patients to assess who may benefit from the procedure.

**Supplementary Information:**

The online version contains supplementary material available at 10.1007/s10151-023-02853-8.

## Introduction

In 1986, Heald and colleagues from Basingstoke revolutionised the outcomes of rectal cancer surgery by reporting total mesorectal excision (TME) [1]. This standardized technique combines proctectomy with complete removal of the mesorectum following the embryological planes, therefore allowing a complete lymphadenectomy of the first lymph node stations, and led to major improvements in overall survival (OS) and local recurrence [2].

In 2009, Hohenberger proposed applying a similar approach to right-sided colon cancer and introduced the concept of complete mesocolic excision (CME). The aim was to improve oncological outcomes by following the hypothesis that an increased number of harvested lymph nodes and complete removal of the mesocolon correlates with improved survival [3].

It is noteworthy that Yamaoka et al. reported that the incidence of lymph node metastases in patients with right-sided colon cancer was 41.5%. These lymph node metastases were present in the D2 lymph nodes in 9.7% of patients and in D3 lymph nodes in 1.5% of patients [4]. However, D3 lymph nodes located along the superior mesenteric vein are not removed when performing conventional (D2) right hemicolectomy. Therefore, to improve the lymph node yield and remove these nodes, CME is based on three concepts: 1. Sharp embryological plane dissection to completely remove the mesocolon, including all the lymph nodes draining the tumour, 2. Central vascular ligation to remove central lymph nodes and 3. Sufficient bowel resection to be able to remove the pericolic lymph nodes and the adjacent vascular arcades [5, 6]. This partially overlaps with the recommendation from Asian professional societies, notably the Japanese Society for Cancer of the Colon and Rectum, which recommends D3 lymphadenectomy (LND3) for ≥ cT2 colorectal cancer [7].

A recent consensus statement article by Tejedor et al. showed that the main characteristics of the procedure to qualify for CME are central vascular ligation, exposure of the superior mesenteric vein and intact mesocolon excision [8].

Complete mesocolic excision could therefore theoretically improve overall and disease-free survival for selected patients with right-sided colon cancer. However, implementation of this procedure has not been universally adopted, because doubts have been raised concerning a potential increase in the perioperative morbidity of the procedure, notably central vascular injuries, and also as a result of its increased technical difficulty. Therefore, we aimed to assess the current state of the literature to determine if CME in right hemicolectomy has better oncological outcomes when compared to non-CME procedures and if it has an impact on perioperative morbidity/mortality.

## Methods

The present methodology follows the Preferred Reporting Items for Systematic Reviews and Meta-Analyses (PRISMA) 2020 guidelines [9]. The study protocol was registered in the PROSPERO database (CRD42021225411).

### Literature search

Medline, Embase, and Web of Science were searched on 22 February 2023. The literature search strategy is reported in Table 1. Additional records were identified by screening the reference lists of existing reviews in the field.

**Table 1:** Literature search strategy

Web of science	AB = ((complete mesocolic excision OR CME) AND (colectomy OR hemicolectomy OR colon resection) AND (cancer)) and Article (Document Types) and 2000–2023 (Publication Years)
Pubmed/Medline	((Complete Mesocolic Excision [Title/Abstract]) OR (CME [Title/Abstract]) OR (Mesocolon [MeSH Terms)) AND ((colectomy[MeSH Terms] AND cancer[MeSH Terms])) Filters applied: Adult: 19+ years, 2000–2023
Embase	(‘complete mesocolic excision’:ab,ti OR ‘cme’:ab,ti) AND (‘colectomy’:ab,ti OR ‘hemicolectomy’:ab,ti OR ‘colon resection’:ab,ti) AND (‘cancer’:ab,ti OR ‘malignant neoplasia’:ab,ti) AND ‘article’/it AND [2000–2023]/py

### Study selection: inclusion and exclusion criteria

In this article, we use the term CME to define the technique reported by Hohenberger et al. [6]. Conversely, we use the term non-CME to designate the study control groups defined by the use of standard right hemicolectomy. D2 lymphadenectomy (LND2), the conventional procedure used in most centres in our studies, is defined by the Japanese Society for Cancer of the Colon and Rectum and requires the removal of the pericolic and intermediate lymph nodes [10]. To be included, publications had to be original studies reporting on short- and long-term oncological and non-oncological outcomes of adult patients undergoing right hemicolectomy with CME as a curative treatment for primary colorectal cancer. Studies including fewer than 50 patients, those which included patients with metastatic disease, those without a control group (with non-CME right hemicolectomy), the grey literature, or any kind of secondary analysis (meta-analyses, systematic reviews, or duplicate patients) were excluded. No language restriction was applied.

### Data extraction and outcomes

Two reviewers (GDL, JM) independently selected articles for inclusion and extracted the data according to a pre-established data collection form. Any discrepancies were solved by reaching a consensus between the two reviewers. The following data were extracted: first author, publication year, the country where the investigation took place, study period, study design, TNM inclusion criteria, number of patients, number of patients who underwent CME, sex, age, surgical technique, postoperative pain, time to passage of flatus, time to diet, blood loss, number of harvested lymph nodes, length of hospital stay, postoperative complications, conversion rate, reoperation rate, 30-day mortality, 3-year and 5-year OS, 3-year and 5-year disease-free survival (DFS), local recurrence, metastatic recurrence, and overall recurrence.

### Risk of bias assessment of included studies

The Cochrane risk of bias tool was used to assess the quality and risk of bias for the included randomised controlled study [11]. The methodological quality of all included non-randomised studies was assessed on the basis of the Newcastle–Ottawa Scale [12]; the studies that scored ≥ 7 were considered to be of high quality. This tool was used to assess each study for eight parameters and categorised into three groups: first, the selection of the study groups; second, the comparability of the groups; and third, the ascertainment of either the exposure or outcome of interest for case–control studies. One point was awarded for each quality item. High-quality studies were awarded up to 9 points [12] (Table 1). Funnel plots were used to check the risk of publication bias (Supplemental material 1).

### Statistical analysis

Qualitative data were summarized in tables. For studies that only provided median and interquartile range, conversion into mean was done using the method proposed by Liu et al. [13] and standard deviation with the method from Wan et al. [14]. Quantitative analysis was performed using the ReviewManager 5.4.1 software (The Cochrane Collaboration, The Nordic Cochrane Center, Copenhagen, Denmark). Odds ratios with 95% CI were reported. Model with random effects was applied (DerSimonian and Laird’s approach). Heterogeneity was quantified using the *I*^2^ value. The results of meta-analyses are shown as forest plots. Statistical significance was defined as *p* < 0.05.

## Results

### Study selection process

**Fig. 1:**
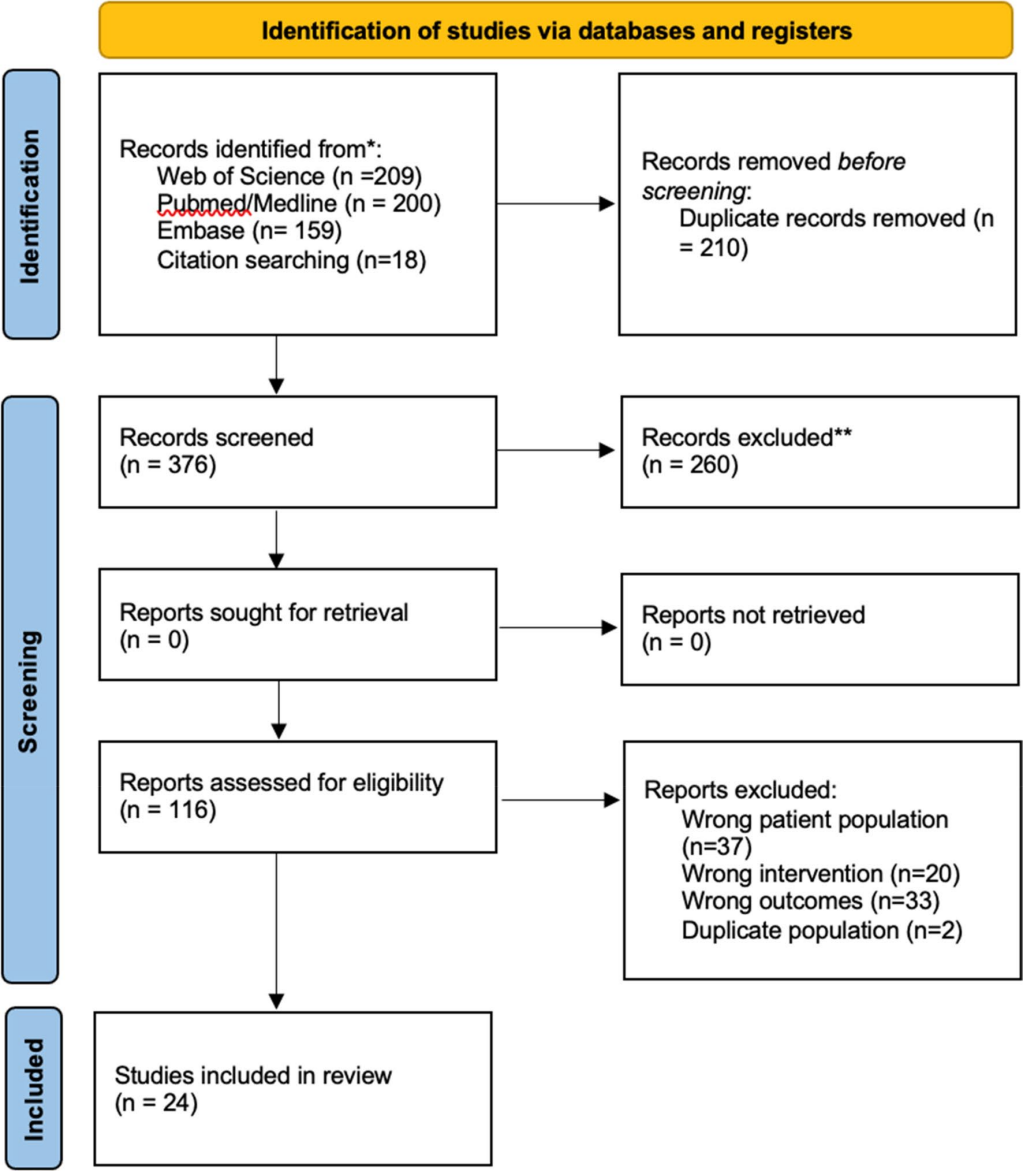
PRISMA flow chart of the inclusion process

A total of 209 publications were identified on Web of Science, 200 on Pubmed/Medline, and 159 on Embase. Eighteen publications were identified from citation searching. Two hundred and ten duplicates were removed. Of the 376 publications that were identified as eligible, 260 were excluded after title/abstract screening and 92 after the full-text screening, based on our inclusion/exclusion criteria. Ultimately, 24 publications were included in the qualitative and quantitative review (Fig. 1).

### Characteristics of included studies

Included studies were conducted in Europe and Asia. These articles were recent; 15 were published in the last 5 years [15–29], and none before 2013. Data from three studies were prospectively collected [17, 24, 28], two were retrospective case–control studies [15, 16], 18 were retrospective cohort studies [18, 19, 21–23, 25–27, 29–38], and one was a randomised controlled trial [20]. Thirteen compared surgical techniques for CME [25–29, 31–38]. All studies included patients with right colon cancer stages T1 to T4, N0 to N2, and without any known distant metastases. TNM stages ranged between I and III. Three studies included only laparoscopic surgery [15, 19, 20], and one study only open surgery [30]. Newcastle–Ottawa Scale scores ranged between 6/9 and 8/9. Cochrane Risk of Bias assessment for our included randomised controlled trial showed a high risk of bias for blinding of participants and personnel, low risk of bias for random sequence generation, blinding of outcome assessment, selective outcome reporting and other sources of bias, and an unclear risk of bias for allocation concealment and incomplete outcome data. Data from studies comparing CME vs non-CME are summarized in Table 2.

**Table 2:** Characteristics of included studies comparing CME versus non-CME

Study	Country of the first author	Study period	Type of study	TNM stage (inclusion)	Patients,* n*	CME patients, *n* (%)	Male (%)	Age, years	Surgical technique, *n*	Newcastle–Ottawa Scale (score)
An, 2018	South Korea	08.2007–10.2011	Case–control	I–III	115	34 (29.6)	50.6	< 70 (mean)	Laparoscopic (115)	7/9
Benz, 2013	Germany	01.2005–12.2010	Retrospective cohort	I–III	107	69 (65.1)	55.7	< 70 (CME), > 70 (non-CME) (mean)	Open (107)	8/9
Bernhoff, 2018	Sweden	01.2004–12.2012	Case–control	I–III	709	236 (33.3)	54.2	> 70 (median)	Laparoscopic (−), open (−)	7/9
Bertelsen, 2019	Denmark	06.2008–12.2013	Prospective cohort	I–III	1069	256 (23.9)	41.9	> 70 (median)	Laparoscopic (604), open (465)	8/9
Bertelsen, 2018	Denmark	06.2008–12.2013	Retrospective cohort	I–III	465	141 (30.3)	43.9	> 70 (median)	Laparoscopic (290), open (175)	8/9
Ouyang, 2019	China	01.2008–12.2010	Retrospective cohort	I–III	167	107 (64.1)	57.4	< 70 (mean)	Laparoscopic (167)	8/9
Xu, 2021	China	01.2016–12.2019	Randomised controlled trial	I–III	995	495 (49.7)	58.8	< 70 (median)	Laparoscopic (995)	–
Giani, 2022	Italy	01.2010–12.2018	Retrospective cohort	I–III	292	146 (50.0)	44.8	> 70 (median)	Laparoscopic (264), open (28)	8/9
Tümay, 2020	Turkey	02.2006–06.2019	Retrospective cohort	I–III	87	48 (55.2)	42.5	< 70 (mean)	Laparoscopic (14), open (73)	6/9
Khan, 2021	UK	2007–2017	Retrospective cohort	I–III	120	40 (33.3)	46.7	< 70 (CME), > 70 (non-CME) (median)	Robotic (40), laparoscopic (80)	7/9
An, 2018	South Korea	08.2007–10.2011	Case–control	I–III	115	34 (29.6)	50.6	< 70 (mean)	Laparoscopic (115)	7/9

### Short-term outcomes

The data on short-term outcomes are summarized in Table 3.

**Table 3:**
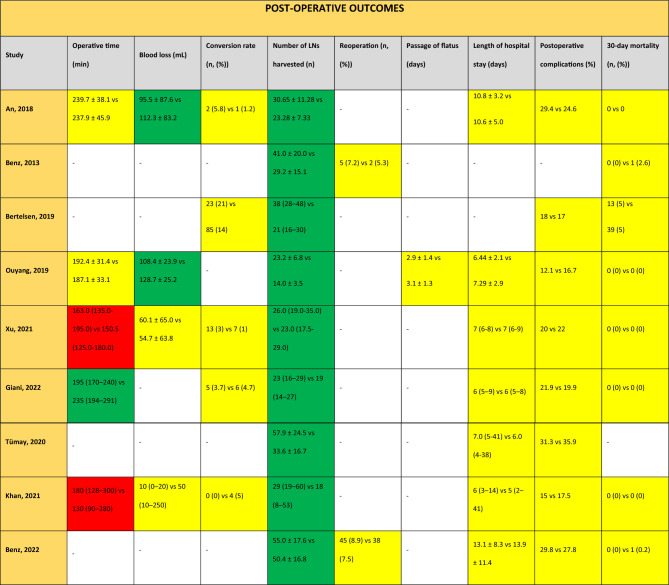
Postoperative outcomes for studies comparing CME versus non-CME

Green in favour of CME (*p* value < 0.05), yellow neutral (*p* value > 0.05), red in favour of non-CME (*p* value < 0.05), and white not reported. The value reported first corresponds to results for CME and the second value for non-CME. Values are reported as mean ± standard deviation or median (range) LN =Lymph node

#### Operative time

**Fig. 2:**
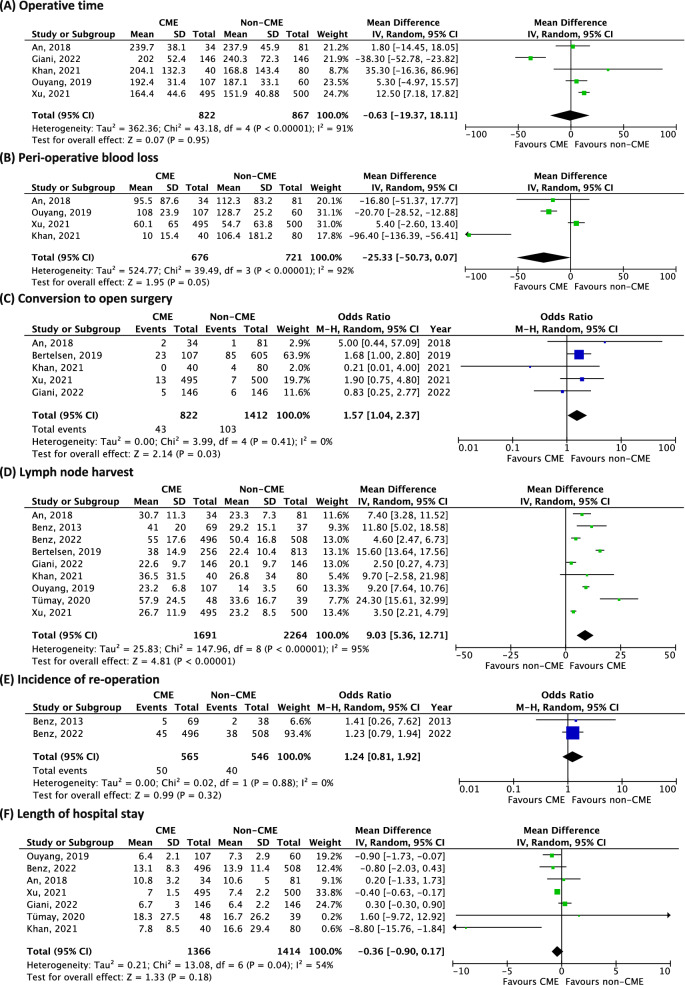

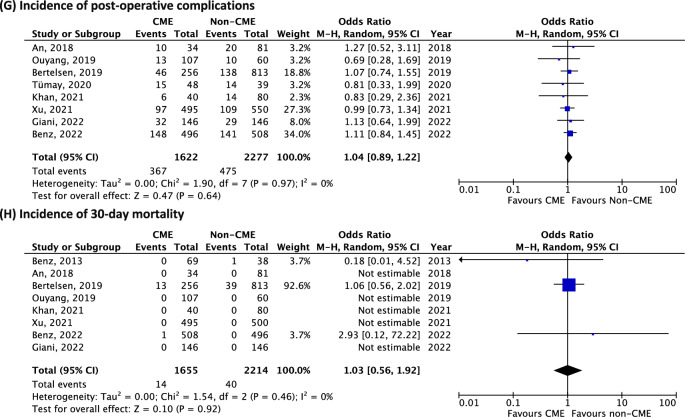
Meta-analysis of CME versus non-CME. Forest plots of short-term outcomes comparing CME versus non-CME. Each horizontal bar summarizes a study. The bars represent 95% confidence intervals. The squares inform each study’s weight in the meta-analysis. The diamond in the lower part of the graph depicts the pooled estimate along with 95% confidence intervals. Odds ratio (OR) and mean difference (MD) were obtained using models with random effect (Mantel–Haenszel). Heterogeneity was assessed using the Q-test and quantified using the *I*^2^ value

Two studies reported no significant difference in terms of operative time [15, 19], two studies reported an increased operative time for the CME group [20, 23], and one reported a decrease in operative time for the CME group [21]. In the quantitative meta-analysis, no difference was found in terms of mean operative time between the CME and the non-CME group (5 studies, 1689 patients, mean difference (MD) − 0.63, 95% confidence interval (CI) − 19.37–18.11, *p* = 0.95, *I*^2^ 91%) (Fig. 2a).

#### Perioperative blood loss

Two studies reported a statistically significant decrease in blood loss in the CME group [15, 19]. Two other studies reported no significant difference in this outcome [20, 23].

According to our meta-analysis, there was no difference in mean blood loss between the CME and the non-CME group (4 studies, 1397 patients, MD − 25.33, 95% CI − 50.73–0.07, *p* = 0.05, *I*^2^ 92%) (Fig. 2b).

#### Conversion to open surgery

Five studies reported the incidence of conversion to open surgery. The incidence of conversion to open surgery ranged between 1% and 14% for the non-CME group [17, 20], and between 0% and 21% for the CME group [17, 20]. Meta-analysis found that patients who underwent CME were at increased risk of conversion to open surgery (5 studies, 2234 patients, OR 1.57, 95% CI 1.04–2.37, *p* = 0.03, *I*^2^ 0%) (Fig. 2c).

#### Lymph node harvest

All studies comparing CME versus non-CME reported a statistically significant increase in harvested lymph nodes in favour of the CME technique, with mean values ranging between 22.6 and 57.9 lymph nodes for the CME group and between 14.0 and 50.4 lymph nodes for the non-CME group [15, 17, 19–24, 30]. Meta-analysis found that CME increased the number of harvested lymph nodes by 9 lymph nodes when compared to non-CME (9 studies, 3955 patients, MD 9.03, 95% CI 5.36–12.71, *p* < 0.00001, *I*^2^ 95%) (Fig. 2d).

#### Incidence of reoperation

Two studies reported the incidence of reoperation, which ranged between 5.3% and 7.5% for the non-CME group, and between 7.2% and 8.9% for the CME group. These differences were not statistically significant [24, 30]. Pooled analysis did not show any difference in terms of the incidence of reoperation between the CME and the non-CME group (2 studies, 1111 patients, OR 1.24, 95% CI 0.81–1.92, *p* = 0.32, *I*^2^ 0%) (Fig. 2e).

#### Time to first passage of flatus

One study reported time to passage of flatus, with a mean value of 3.1 days (non-CME) versus 2.9 days (CME); this difference was not statistically significant [19].

#### Length of hospital stay

Seven studies reported the length of hospital stay, without showing a statistically significant difference between the techniques: CME groups ranged between 6.4 and 13.1 days, and non-CME groups ranged between 6.4 and 16.7 days [15, 19–24]. Quantitative analysis did not show any difference between CME and non-CME in terms of length of hospital stay (7 studies, 2780 patients, MD − 0.83, 95% CI − 0.9–0.17, *p* = 0.18, *I*^2^ 54%) (Fig. 2f).

#### Incidence of postoperative complications

Eight studies reported similar incidences of postoperative complications such as anastomotic leak, chylous ascites, pneumonia, bleeding, small bowel obstruction, wound infection, venous thromboembolism and urinary tract infections between CME (incidence ranging between 12.1% and 31.3%) and non-CME (incidence ranging between 16.7% and 35.9%) [15, 17, 19–24]. One study reported a median follow-up for postoperative complications of 30 days [20]. One study followed patients for complications for 60 days [17], and another study for 90 days [21]. The timing for follow-up was not reported in 5 studies [15, 16, 19, 22, 24]. According to the meta-analysis, there was no difference in terms of the incidence of postoperative complications between the CME and the non-CME group (8 studies, 3899 patients, OR 1.04, 95% CI 0.89–1.22, *p* = 0.97, *I*^2^ 0%) (Fig. 2g). The funnel plot for this outcome shows a symmetrical distribution (Supplemental material 1A).

#### Incidence of 30-day mortality

The incidence of 30-day mortality was reported in eight articles ranging between 0% and 5% for both the CME and the non-CME groups [17, 19–21, 23, 24, 30], with no significant difference. Quantitative analysis did not show any difference in terms of 30-day mortality rates between the CME and the non-CME group (8 studies, 3869 patients, OR 1.03, 95% CI 0.56–1.92, *p* = 0.92, *I*^2^ 0%) (Fig. 2h).

### Long-term outcomes

The data on long-term outcomes is summarized in Table 4.

**Table 4:**
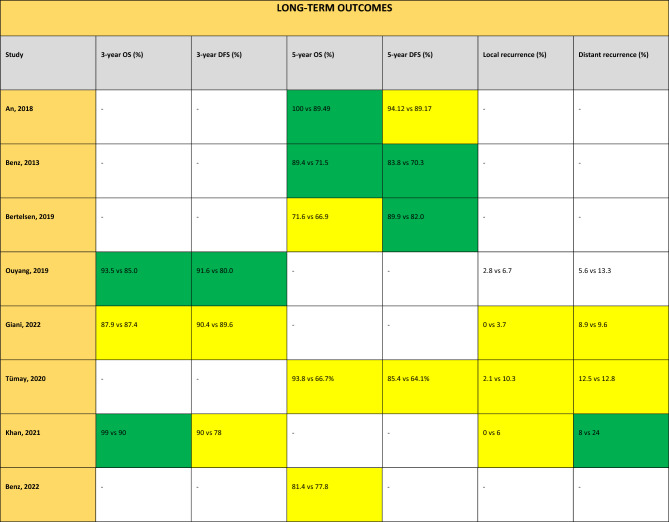
Long-term outcomes of interest for studies comparing CME versus non-CME

Green in favour of CME (*p* value < 0.05), yellow neutral (*p* value > 0.05), red in favour of non-CME (*p* value < 0.05), and white not reported. The value reported first corresponds to results for CME and the second value for non-CME. *LN* lymph node, *OS* overall survival, *DFS* disease-free survival, *LR* local recurrence, *MR* metastatic recurrence

### Three-year overall survival

Two studies reported an increase in 3-year OS: Ouyang et al. reported 93.5% (CME) versus 85.0% (non-CME) (*p* = 0.017) [19], and Khan et al. reported 99% (CME) versus 90% (non-CME) (*p* = 0.0454) [23]. One other study reported no significant difference [21].

**Fig. 3:**
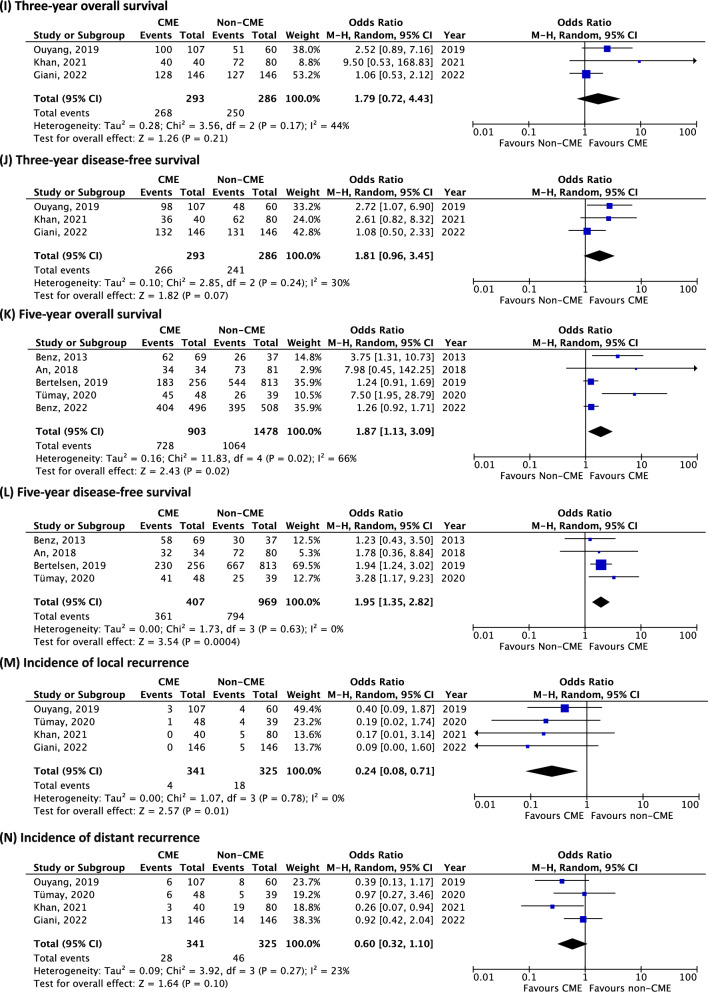
Meta-analysis of CME versus non-CME. Forest plots of long-term outcomes comparing CME versus non-CME. Each horizontal bar summarizes a study. The bars represent 95% confidence intervals. The squares inform each study’s weight in the meta-analysis. The diamond in the lower part of the graph depicts the pooled estimate along with 95% confidence intervals. Risk ratio (RR) was obtained using models with random effect (Mantel–Haenszel). Heterogeneity was assessed using the Q-test and quantified using the *I*^2^ value

In the quantitative meta-analysis, no difference was found in terms of 3-year OS between the CME and the non-CME group (3 studies, 579 patients, OR 1.79, 95% CI 0.72–4.43, *p* = 0.21, *I*^2^ 44%) (Fig. 3i).

### Three-year disease-free survival

Three-year DFS was greater in the CME group in one study, with a reported 91.6% (CME) versus 80.0% (non-CME) (*p* = 0.014) [19]. Two studies reported no significant difference [21, 23].

Pooled analysis did not show any difference in terms of the incidence of 3-year DFS between the CME and the non-CME group (3 studies, 579 patients, OR 1.81, 95% CI 0.96–3.45, *p* = 0.7, *I*^2^ 30%) (Fig. 3j).

### Five-year overall survival

Two studies reported an increase in 5-year OS for the CME group: 100% (CME) versus 89.49% (non-CME) (*p* = 0.049) [15], and 89.4% (CME) versus 71.5% (non-CME) (*p* = 0.011) [30]. Three studies reported no difference between the two groups for this outcome [17, 22, 24].

Meta-analysis found that patients who underwent CME showed improved 5-year OS (5 studies, 2381 patients, OR 1.87, 95% CI 1.13–3.09, *p* = 0.02, *I*^2^ 66%) (Fig. 3k). The funnel plot for this outcome shows an asymmetrical distribution, indicating potential publication bias (Supplemental material 1B).

#### Five-year disease-free survival

Five-year DFS was similar for both groups in two studies [15, 22], but two studies reported results in favour of the CME group: 93.8% (CME) versus 78% (non-CME) (*p* = 0.049) [30], and 89.9% (CME) versus 82.0% (non-CME) (*p* = 0.00028) [17].

Pooled analysis showed an improvement in terms of 5-year DFS for the CME group (4 studies, 1376 patients, OR 1.95, 95% CI 1.35–2.82, *p* = 0.0004, *I*^2^ 0%) (Fig. 3l). The funnel plot for this outcome a shows symmetrical distribution (Supplemental material 1C).

#### Incidence of local recurrence

All four studies reporting incidence of local recurrence showed no significant difference [19, 21–23].

Quantitative analysis showed improved incidence in terms of local recurrence for the CME group (4 studies, 666 patients, OR 0.24, 95% CI 0.08–0.71, *p* = 0.01, *I*^2^ 0%) (Fig. 3m).

#### Incidence of distant recurrence

One study reported an improved incidence in distant recurrence, with 8% (CME) versus 24% (non-CME) (*p* = 0.026) [23], whilst three studies no significant difference for this outcome [19, 21, 22].

Pooled analysis showed no difference in the incidence of distant recurrence between the CME and the non-CME group (4 studies, 666 patients, OR 0.60, 95% CI 0.32–1.10, *p* = 0.10, *I*^2^ 23%) (Fig. 3n).

## Discussion

Studies comparing CME and non-CME included in this review have tended to show that CME increases the number of lymph nodes harvested, without increasing postoperative complications, or 30-day mortality. The incidence of conversion to open surgery seemed to be increased in the CME group, but this may be due to one included study where authors reported higher overall conversion rates as a result of the implementation of laparoscopic surgery in their centres [17]. The ongoing randomised controlled trial included in our review, named the RELARC trial, has for the moment only published preliminary short-term outcomes but will release long-term outcomes in the future. The authors concluded that CME did not increase perioperative complications of right hemicolectomy compared to non-CME but, when looking at the detailed results, the incidence of vascular injury was significantly increased (3% for CME versus 1% for non-CME, *p* < 0.05) [20]. A randomised clinical trial in Russia comparing short-term outcomes for CME versus non-CME (COLD) showed no difference in mortality or complication rates [39], and another prospective non-randomised controlled trial in China showed no difference in perioperative morbidity/mortality [40]—these studies did, however, include both left- and right-sided resections. As CME is technically challenging to perform, favourable short-term outcomes depend on the experience and technique of the surgical team, as shown by Bernhoff et al. [16].

Regarding long-term outcomes, our meta-analysis showed improved local recurrence rates and an increase in 5-year OS and 5-year DFS. Bertelsen et al. also reported a risk reduction in 5-year OS, but only in their 2010–2013 subgroup, which suggests the impact of a learning curve for this procedure. They also showed risk reduction in 5-year DFS, especially for the higher stages, and once again more marked in the most recent subgroup [17]. The recent prospective study by Benz et al. which included 1004 patients has shown no significant increase in 5-year OS. However, in patients with stage III disease, the authors did report a significant increase in this outcome (78.3% CME versus 65.0% non-CME). It is also worth mentioning that this study included pathological specimen control by mesenteric area to further determine if the technique was successfully performed [24]. A prospective study by Gao et al. showed improved 3-year local recurrence-free survival (100% CME vs 90.2% non-CME) but did include both left- and right-sided disease [40].

Long-term impact on bowel function was assessed by Bertelsen et al., who did not find an increased risk of bowel dysfunction, long-term pain or impaired quality of life when compared to non-CME [18].

Recent data concerning preoperative staging have shown that computed tomography lacks accuracy, especially in predicting lymph node metastases, with a sensitivity of 57% and a specificity of 66%. This has an impact on disease management in general but also on the potential choice of surgical technique [41].

To our knowledge, CME versus non-CME for right hemicolectomy had previously been analysed in several systematic reviews with meta-analyses, the most recent ones being published in 2021. In their analysis, Balciscueta et al. included 29 studies and obtained a complication rate of 17.4% (CME group) versus 19.4% (non-CME) (*p* = 0.84), and a 5-year OS of 78.2% (CME) versus 67.1% (non-CME) (*p* = 0.03). They subclassified OS according to stage II and stage III disease, which showed a greater advantage for the CME group [42]. De Simoni et al. included 8 studies in their meta-analysis and obtained no difference between techniques for complication rates (odds ratio 1.13, *p* = 0.34). However, they showed a significant benefit in terms of 3-year OS (odds ratio 1.57, *p* = 0.003) and 5-year OS (odds ratio 1.41, *p* = 0.02) in favour of CME [43]. Anania et al. included 17 studies in their meta-analysis and obtained no difference between techniques for complication rates (relative risk 0.82, CI 0.67–1.00). However, they showed a significant benefit in terms of 3-year OS (relative risk 0.42, 95% CI 0.27–0.66) and 5-year OS (relative risk 0.36, 95% CI 0.17–0.56) in favour of CME [44]. Díaz-Vico et al. included 27 studies and reported a higher 3-year OS (relative risk 1.09, *p* = 0.01) and 5-year OS (relative risk 1.05, *p* = 0.02) for CME, without any increase in the complication rate (relative risk 1.13, *p* = 0.58) [45]. Ferri et al. included 17 studies and reported a complication rate of 21% (CME group) versus 24% (non-CME) (*p* = 0.66), 5-year DFS of 81.4% (CME) versus 79.7% (non-CME) (*p* < 0.01), and a 5-year OS of 86% (CME) versus 70.8% (non-CME) (*p* < 0.01) [46]. All these meta-analyses of non-randomised studies shared the conclusion that CME improves DFS, OS, and local and metastatic recurrence when compared to non-CME, whilst showing similar postoperative complication rates, which is a trend also shown in our study.

Our systematic review and meta-analysis on short- and long-term outcomes of CME is to our knowledge the first to include only comparative studies with non-metastatic patients specifically undergoing right hemicolectomy. Our review also benefits from a large patient sample, including the most recent studies. Since the last systematic review/meta-analysis, we have identified four new cohort studies and one new randomised controlled trial. The main strength of this review is its thorough evaluation of CME in terms of short- and long-term outcomes.

Conceptually, CME is similar to another procedure used since 1977 in Asia, notably in Japan: right hemicolectomy with LND3. Although conceptually similar, there are a few differences between LND3 and CME. First, CME involves strict embryological plane dissection rather than removing central lymph node stations, which sometimes involves the removal of the lymph nodes around the head of the pancreas or the gastroepiploic arcade. Second, LND3 requires at least 10 cm resection margins, but CME is usually more extensive because of the requirement to remove adjacent vascular arcades. Third, LND3 usually spares the root of the main colic vessels because the ligation only occurs at the root of the primary feeding vessels, whereas CME mandates more radical central vascular ligation and dissection up to the root of the main colic vessels [47].

The main limitations of this review are the following: first, data on right hemicolectomy with CME are still scarce and not of the highest quality; second, almost all included studies are cohort studies, which may carry a risk of selection bias; third, publication bias may be present; and finally, the quality evaluation of the pathological specimens is heterogeneous and not always reported. This makes it difficult to correctly assess if the patient benefited from the maximum effect of CME and, on the other hand, many supposed conventional non-CME procedures may have been more/less extensive than the standard LND2, as has been outlined by Bertelsen et al. reporting suboptimal survival rates in the control group for stage I and II disease [48]. Future randomised controlled trials should include quality control by expert surgeons not only for CME but also for the control group. On this aspect, Siani et al. proposed a classification for pathological evaluation, which could be useful for future research [49, 50]. It is also worth noting that surgeons practising CME are usually more experienced and can therefore report lower incidences of postoperative complications—this raises a word of caution as the widespread implementation of the technique may lead to an increase in postoperative morbidity and severe complications including central vascular injuries. Finally, the use of (neo-)adjuvant therapy and other important variables were not systematically reported, which could introduce a bias in terms of survival outcomes. It is also worth noting that the biology of colorectal cancer is still not fully understood and that survival is not solely based on lymph node involvement—other factors, such as extramural venous invasion may also play a role in the prognosis of the disease [51]. This review also does not take into account the emerging role of immunotherapy in digestive surgery [52].

The fact that improved lymphadenectomy appears to improve survival has classically been attributed to the stage migration phenomenon, but some authors are suggesting this may also be a reflection of molecular features of the cancer [53]. This bias may also have been present in our included studies. Detection bias is limited by homogenous and standardized follow-up, which is reported in most included studies.

As outlined, the ongoing RELARC trial will soon be publishing long-term survival results. These authors are waiting to release long-term results to determine if survival rates could be improved, which would constitute a justification for the increased complexity of the CME technique [20]. A multicentre randomised prospective study comparing CME versus non-CME is currently recruiting in Italy (CoME-IN) and will report short- and long-term outcomes [54], and a prospective multicentre cohort study in South Korea is evaluating the oncologic outcomes of laparoscopic modified CME (PIONEER).

## Conclusion

Current pooled evidence of observational and randomised studies suggests that CME for right hemicolectomy improves the lymph node yield, 5-year OS and 5-year DFS when compared to non-CME, with similar perioperative morbidity. Similarly, the incidence of recurrence is decreased. However, a recent randomised controlled trial, pooled in our meta-analysis, questions the incidence of perioperative complications, and showed a significant incidence of central vascular injuries. These results require confirmation by high-quality evidence, such as multicentre randomised controlled trials with long-term follow-up.

### Supplementary Information

Below is the link to the electronic supplementary material.

Supplementary file1 (DOCX 53 KB)

## Data Availability

All data generated or analysed during this study are included in this published article.
