# Supplementary material for: Complete mesocolic excision for right hemicolectomy: an updated systematic review and meta-analysis
Source: Tech Coloproctol. 2023 Aug 26;27(11):979–93. doi: 10.1007/s10151-023-02853-8 (PMC10562294; doi:10.1007/s10151-023-02853-8)
Supplement: Supplementary file 1 — Supplementary file1 (DOCX 53 KB) [file 10151_2023_2853_MOESM1_ESM.docx]

**(A) Post-operative complications**

**(B) 5-year OS**

**(C) 5-year DFS**
